# Approaching the holistic transcriptome—convolution and deconvolution in transcriptomics

**DOI:** 10.1093/bib/bbaf388

**Published:** 2025-08-03

**Authors:** Maik Wolfram-Schauerte, Thomas Vogel, Hanati Tuoken, Maria Fälth Savitski, Eric Simon, Kay Nieselt

**Affiliations:** Faculty of Science, Department of Computer Science, Eberhard-Karls University Tübingen, Sand 14, D-72076 Tübingen, Baden-Württemberg, Germany; Faculty of Science, Department of Computer Science, Eberhard-Karls University Tübingen, Sand 14, D-72076 Tübingen, Baden-Württemberg, Germany; Computational Innovation, Boehringer Ingelheim Pharma GmbH & Co. KG, Birkendorfer Str. 65, D-88397 Biberach, Baden-Württemberg, Germany; Computational Innovation, Boehringer Ingelheim Pharma GmbH & Co. KG, Birkendorfer Str. 65, D-88397 Biberach, Baden-Württemberg, Germany; Computational Innovation, Boehringer Ingelheim Pharma GmbH & Co. KG, Birkendorfer Str. 65, D-88397 Biberach, Baden-Württemberg, Germany; Faculty of Science, Department of Computer Science, Eberhard-Karls University Tübingen, Sand 14, D-72076 Tübingen, Baden-Württemberg, Germany

**Keywords:** convolution, deconvolution, bulk RNA-Seq, scRNA-seq, transcriptomics, machine learning, holistic transcriptome, cell-type proportions

## Abstract

Tissues, organs, and entire organisms are composed of diverse cell populations, which are characterized by cell-type-specific gene activities. Bulk RNA-seq represents a robust, cost-effective, scalable method to measure gene activity at the bulk tissue level. However, pathomolecular processes lead to divergent changes in tissue composition and cell-type-specific gene deregulations, which cannot be resolved at the tissue bulk level without information on either change in cell-type proportion or expression at the single-cell level. Accordingly, methods have been developed that constrain bulk deconvolution by information from single-cell expression or cell-type proportion. In parallel, convolution methods have been developed to project single-cell expression to bulk tissue level (pseudobulk simulation). In the present review, we provide an overview of existing convolution and deconvolution methods, their interconnectivity, and benchmarking. Our unique approach lies in the joint consideration of both directions in a “holistic transcriptome model.” Through analysis of published (de)convolution studies and benchmarks, we identified the reduced availability of suitable datasets and the use of inaccurate convolution-like methods for (de)convolution model assessment and training as key bottlenecks in the field. On that basis, we conclude with a holistic transcriptome model envisioning that a more integral approach to convolution and deconvolution is needed. With our suggestions for a unified framework we aim to spark collaborative efforts to enable major leaps forward in the field of (de)convolution.

## Introduction

Living systems are organized by a diverse array of single cells and their complex interactions that give rise to tissues, organs, and organisms. Disease phenotypes on the bulk tissue scale are contrasted by their molecular cause on the single-cell scale. Therein, not only dysregulation of cellular processes can affect tissue or organ function, but also the cell-type composition.

Changes in **cell-type proportions** (see Glossary in Table 1) in a tissue or organ are considered hallmarks of diseases [1–5]. These changes play a key role in fibrotic diseases (e.g. “epithelial-to-mesenchymal transition”) or inflammatory conditions characterized by immune infiltration, including liver disease [6, 7], cardiovascular disease [8, 9], or neurodegenerative diseases [1, 10]. Also, cancer research is considering not only the increasing abundance of malignant cells during tumor progression, but also the tumor microenvironment (TME; see the list of abbreviations in Table 2) [11, 12]. The TME is an increasingly studied hallmark of cancer, which appreciates that diverse (immune) cells such as tumor-associated macrophages can influence tumor growth and thus disease progression [12]. Consequently, investigating tissues on the single-cell level is crucial for understanding their function and organization and can provide essential insights into disease origin and progression. Hence, knowing the abundance of cell types and their molecular profiles is key to understanding diseases and their pathomechanisms and can inform the identification of biomarkers or treatment targets. Thus, this has the potential to significantly boost drug discovery and unlock (novel) therapeutic targets.

**Table 1:** Glossary—definition of key terms in this article

**Term**	**Description**
Bulk RNA-seq	RNA sequencing after RNA isolation of an entire tissue or (mixed) population of cells
Cell-type deconvolution	Deconvolution of a bulk transcriptome to predict cell type proportions in the bulk
Cell-type proportions (also cell-type fractions)	Relative abundances of cell-types in given tissues
Convolution	Projection of single-cell expression data to bulk expression data given cell type proportions
Deconvolution	Decomposition of bulk expression data into cell type proportions or cell type expression profiles
Fluorescence-activated cell sorting (FACS)	Technique for sorting single cells from a dissociated tissue into distinct populations based on fluorescent signals of intrinsic or cell surface markers
Immuno-histochemistry (IHC)	Staining and imaging technique to visualize and identify cells in their spatial context
Matched dataset	A dataset including scRNA-seq/snRNA-seq data, cell type abundance measurements and bulk RNA-seq data from the same tissue sample
Pseudobulk	Simulated bulk expression profile using scRNA-seq data
Single-cell deconvolution	Deconvolution of a bulk transcriptome to obtain a cell type-specific expression profile
Single-cell RNA-seq (scRNA-seq)	RNA sequencing of fresh single cells after tissue dissociation; several platforms and technologies available such as Smart-Seq or 10 x Genomics
Single-nucleus RNA-seq (snRNA-seq)	RNA sequencing of single nuclei isolated also from frozen tissue

**Table 2:** List of abbreviations

**Abbreviation**	**Full description**
ANN	Artificial neural network
CATD	Critical assessment of transcriptomic deconvolution
DE	Differential expression
DWLS	Dampened weighted least squares
FACS	Fluorescence activated cell sorting
GEO	Gene Expression Omnibus
GSEA	Gene set enrichment analysis
IHC	Immunohistochemistry
LDA	Latent dirichlet allocation
MLE	Maximum likelihood estimation
NMF	Nonnegative matrix factorization
NNLS	Nonnegative least squares
Nu-SVR	Nu-support vector regression
OLS	Ordinary least squares
PBMCs	Peripheral blood mononuclear cells
RNA-seq	RNA sequencing
scRNA-seq	Single-cell RNA sequencing
snRNA-seq	Single-nucleus RNA sequencing
TCGA	The Cancer Genome Atlas
TME	Tumor microenvironment
TPM	Transcripts per million

### Study of gene expression and cell-type abundance

Various technologies have been developed and widely applied to study changes in cell-type abundance and gene expression in a tissue or organ of interest.

**Immunohistochemistry** (IHC) or **flow cytometry** are employed as direct cell-type abundance measurement technologies. Despite potential biases, these techniques may accurately determine cell-type composition. In addition, IHC can inform cellular morphology within spatial context of a tissue. However, these methods are comparably low in throughput, are quite costly, can be limited to a predetermined number of cell types of interest, and resulting data can be challenging to analyze [13].

In contrast, **transcriptomics** offer ways to assess tissue complexity via high-throughput gene expression monitoring. In general, one may distinguish three main types of transcriptomics methodologies: bulk, single-cell/single-nucleus, and spatial transcriptomics. In this review, we focus only on bulk and single-cell/(single-nucleus) transcriptomics. Readers interested in spatial transcriptomics technology and (de)convolution in this context may find recent literature informative [14–16].

**Bulk RNA-sequencing** (RNA-seq) monitors the gene expression of (heterogeneous) tissue (i.e. composed of various cell types) at the RNA level. Bulk RNA-seq is the workhorse of gene expression analysis these days. It is standardized, accessible, cost-efficient, and can be applied in high-throughput, scaled to hundreds or thousands of samples per experiment and routinely applied to preserved biospecimen [17]. Consequently, bulk RNA-seq datasets are vastly available in public databases, partially produced by large research consortia [18, 19]. Nevertheless, given that the gene expression of diverse cell types is thereby aggregated in a bulk expression profile, cell-type-specific gene expression and abundance information are convoluted within bulk RNA-seq data [20, 21].

**Single-cell RNA-seq** (scRNA-seq) and **single-nucleus RNA-seq** (snRNA-seq) have emerged as sequencing technologies compensating for this drawback [22, 23]. Essentially, single cells (from blood- or dissociated tissue-derived suspensions) or nuclei (from cryopreserved tissue samples) are isolated and their transcriptomes are monitored with sensitive RNA-seq technologies. This allows hundreds to thousands of single cells from the same or different samples to be sequenced in parallel, depending on the protocol used [22, 23]. Cell-type-specific gene expression profiles, cell-type subpopulations, and gene expression networks can be identified using sc/snRNA-seq demonstrating its enormous potential for biomedical research [24, 25]. Yet, single-cell transcriptomics remains costly and is usually not suitable for defining cell-type proportions, as distinct cell types are recovered with different efficiencies [26, 27]. Also, it is challenging to capture all cell types in a tissue with reasonable coverage and quality. For instance, neutrophils or hepatocytes can be difficult-to-capture cell types, especially regarding their true abundance in the tissue, often requiring specialized sample preparation procedures or multiple single-cell omics measurements (e.g. snRNA-seq and scRNA-seq) [28, 29].

### A connection between single-cell and bulk transcriptomics

Consequently, technologies that measure the abundances of cell types in high-throughput and cost-efficiently are lacking. To this end, about 15 years of past research have aimed at dissecting bulk transcriptome data to determine cell-type abundances—referred to as **deconvolution**. Specifically, deconvolution makes use of machine learning methods to dissect bulk transcriptomes into the underlying cell-type proportions and/or cell-type expression profiles of the corresponding bulk tissue. With the development of single-cell sequencing, techniques for simulation of bulk expression profiles have emerged that are—among others—used to train, test, and benchmark deconvolution methods. **“Convolution,”** on the other hand, refers to the generation of bulk expression profiles from single-cell or cell-type expression data, e.g. from sc/snRNA-seq data, using actual cell-type proportions of the bulk tissue.

Here, we review existing (machine learning) models and algorithms to approach convolution and deconvolution in the field of transcriptomics to study tissue composition. We thereby shed light onto the diverse existing deconvolution tools alongside state-of-the-art convolution methods and their interplay. Importantly, we focus on (de)convolution within bulk and single-cell transcriptomics and do not review aspects of spatial transcriptomics and deconvolution tools. We summarize how (de)convolution tools are benchmarked; identify, evaluate, and list suitable datasets of paired bulk and sc/snRNA-seq; and highlight challenges and chances for the fields of (de)convolution. On that basis, we propose a framework for a “holistic transcriptome model” that takes into account both convolution and deconvolution.

## (De)convolution connects transcriptomics and cell-type abundance modalities

**Figure 1:**
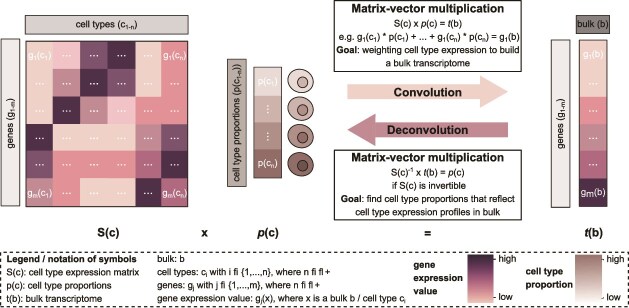
**The holistic transcriptome model—convolution and deconvolution link transcriptomics modalities.** Functions of cell-type-specific gene expression patterns in the form of a genes x cell types matrix ($S(c)$, i.e. as derived from scRNA-seq) and cell-type proportions as column vector ($p(c)$) are combined into a third function ($\mathit{t}(b)$) describing a corresponding bulk transcriptome expression profile of a single sample. This process is referred to as convolution. Vice versa, using bulk gene expression data and knowing either cell-type gene expression profiles or proportions, the respective missing cell-type-specific information can be inferred from bulk expression data. This is referred to as deconvolution. Convolution and deconvolution can be regarded as matrix-vector multiplication operations using the cell-type proportions vector ($p(c)$) for convolution, or—in an idealistic scenario and if invertible—an inverted cell-type expression matrix for bulk deconvolution.

As introduced above single-cell and bulk gene expression as well as cell-type composition are interconnected biological readouts from different scales. For one, cell-type-specific gene expression profiles can be generated from annotated scRNA-seq data, which—combined with cell-type abundances in a given tissue—can be transformed into a bulk expression profile. In the following text, we describe a simple and idealistic mathematical model for the “holistic transcriptome.” In this holistic transcriptome model, this process can be referred to as convolution (Fig. 1). Therein, cell-type expression profiles and corresponding cell-type proportions can be convolved into a third function describing the corresponding bulk transcriptome (equation 1). Let **S** be an $m\times n$ expression matrix, representing cell types $c$ in columns and genes $g$ (or features) in rows. By $p(c)$ we denote a column vector of cell-type ($c$) proportions. We further denote by $t(b)$ a bulk transcriptome profile derived as 


(1) \begin{align*}& S(c) \cdot p(c)^{T}=t(b)\end{align*}$$

Notably, for correct convolution the column vector here needs to be transposed into a row vector by $p(c)^{T}$. Thus, we consider convolution in this model as a matrix-vector multiplication (Fig. 1). In simple terms, convolution first weights each cell-type expression profile by the cell type’s abundance in the tissue and then builds the sum of these weighted expression profiles to yield a bulk transcriptome profile.

“Deconvolution,” on the other hand, refers to the inverse process—the inference of cell-type proportions (given cell-type gene expression information) or cell-type-specific gene expression profiles (given cell-type proportions) from bulk transcriptomics data (Herein, we refer to deconvolution for the inference of cell-type proportions as “deconvolution” only and otherwise specify cell-type or single-cell deconvolution.) (Fig. 1). Basically, deconvolution tries to find the best possible cell-type proportions that reflect cell-type-specific expression in the bulk transcriptome. Thereby gene expression at tissue and single-cell level is linked within the holistic transcriptome model that bridges cell-type abundance and transcriptomics technologies. Compared with previous efforts that focused primarily on deconvolution, our perspective here considers the bi-directionality of the model including both convolution and deconvolution. This model is based on several important biological assumptions such as

- independence of cell types, i.e. cell types have distinct transcriptional profiles and sufficient marker genes exist per cell type;
- linear additivity of gene expression profiles of cell types, where the proportion of a cell type in a tissue determines its fractional contribution to expression in the bulk [30–32];
- homogeneity of cell-type gene expression assuming that cells of the same type exhibit similar expression profiles.

However, given technical variations between the above-mentioned technologies, straightforward projection between the three components of the holistic transcriptome model poses significant challenges. Costs, limited throughput, and potential bias constrain cell-type abundance technologies [13, 33]. While bulk RNA-seq is the reliable high-resolution technique for gene expression monitoring on tissue level [22, 23], it masks cell-type proportion information. Also, its read-out differs from sc/snRNA-seq technologies. In spite of their enormous power, the resulting sc/snRNA-seq data are usually sparse, characterized by dropouts, noise, and technical (and sample processing) bias toward distinct cell types between sequencing platforms and library technologies. While being strong as stand-alone technologies, their output is not necessarily robustly comparable and convertible, which complicates analysis within and across studies [34–37]. Further, the retrieval of cell-type-specific gene expression profiles is influenced, among others, by limitations of cell-type annotation methods or cell capture during library preparation. Consequently, integrating these data modalities in a holistic transcriptome model is challenging.

## Current strategies and tools for convolution

Convolution (see also Fig. 1) describes the forward direction in the holistic transcriptome model. For this, convolution, given cell-type-specific gene expression profiles and the corresponding cell-type composition of the tissue, aims to construct the bulk expression profile of the same tissue. Current efforts, however, do not perform actual convolution according to this definition but rather simulate so-called pseudobulks, a process referred to as “**pseudobulk** simulation.” For this, actual, experimentally determined cell-type proportions are usually not taken into account, with exceptions considering cell-type abundances from scRNA-seq data [38]. Pseudobulks are most commonly used in differential expression (DE) analysis given scRNA-seq data from multiple individuals. Single-cell expression profiles from the same cell type or individual are aggregated and the resulting pseudobulk profiles are then used for DE analysis across cell types or individuals [39, 40]. Notably, these pseudobulk construction methods do not account for (differences) in cell-type proportions between individuals. Moreover, pseudobulks are used when reducing sparsity or dealing with large cell numbers, for instance in gene regulatory network analysis [41–43].

**Figure 2:**
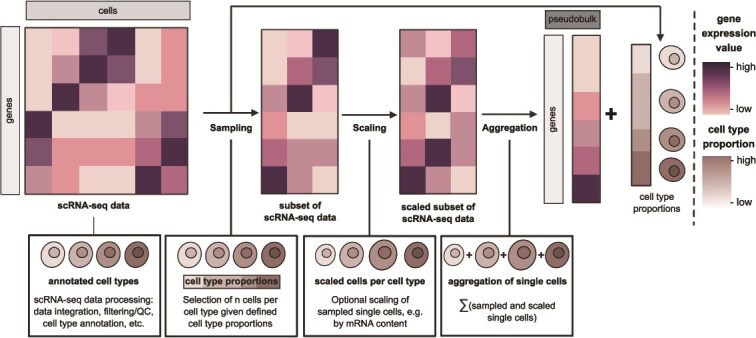
**General concept of the pseudobulk simulation strategy.** Conceptual illustration of pseudobulk simulation, as employed for bulk RNA-seq deconvolution training and testing, as a way of convolution. Single cells are sampled from preprocessed scRNA-seq data based on specified cell-type proportions, optionally scaled to correct for mRNA bias and then aggregated (i.e. summing up transcriptomes of cells) to a pseudobulk profile. Specific cell-type proportions are thereby associated with the pseudobulk sample.

In the context of deconvolution, pseudobulk simulation is used as a method for a different purpose. Typically, single-cell expression data, cell-type proportions, and bulk expression data are rarely available from the same sample, referred to as a “**matched dataset**.” However, they are important to train, test, or evaluate convolution and especially deconvolution methods. Hence, in this context, pseudobulks are generated from scRNA-seq data with user-specified cell-type proportions as associated ground-truth for evaluation [44]. Conceptually, using annotated scRNA-seq data as input, single cells are selected based on their cell type identity according to given proportions of cell types and their expression profiles are subsequently aggregated (Fig. 2). Thereby, the simulated pseudobulk expression profiles are associated with a known ground-truth of cell-type proportions. This approach can provide simulated matched datasets *en masse*, which is vital to provide sufficient training and test data for some deconvolution tools such as Scaden or TAPE [45, 46]. Moreover, simulations allow the generation of bulk data with diverse cell-type compositions and proportions that may offer advantages to model data beyond commonly observed biological variability within tissues. Among others, these enable the assessment of limitations and robustness of deconvolution tools. Pseudobulk simulation may be considered the current state-of-the-art approach to convolution in the described holistic transcriptome model. Notably, the consideration of ground-truth cell-type proportions as defined for convolution in the holistic transcriptome model is usually lacking in pseudobulk simulations. Due to its particular use for deconvolution benchmarking and assessment this will also be considered when reviewing pseudobulk simulation/convolution tools in the following text. We note here that convolution is not extensively mentioned, as it is not widely applied in the field. Rather, pseudobulk simulation is used, which is the approach most closely resembling the principle of convolution aiming to construct a bulk profile from cell-type-specific expression and cell-type proportions.

### Concept of pseudobulk simulation

Overall, the basic strategy for pseudobulk simulation involves the aggregation of single-cell expression profiles that are sampled from a given scRNA-seq dataset to create a pseudobulk expression profile (Fig. 2) [17, 20, 44, 46–48]. This strategy, to our knowledge first applied in 2017 [47], yielded pseudobulks from scRNA-seq data that correlate with actual matched bulk data with Pearson’s correlation coefficients around 0.8 [48]. This correlation is expected not to be much higher due to the different natures of bulk and scRNA-seq library preparation and expression data [48].

After processing and annotation of the scRNA-seq data following best practices [30, 49] (Fig. 2), single cells are sampled from the scRNA-seq dataset. The currently most used pseudobulk simulator **SimBu** [44] offers several options for single cell sampling based on defined cell-type proportions. Among others, these proportions can be customized, randomized, or can reflect the cell-type abundance in the scRNA-seq dataset. Then, selected cells are optionally scaled in the subsequent step (Fig. 2). Single-cell transcriptomes can be captured with varying efficiencies depending on cell types, technical variations, and biases [48]. Various strategies have been employed to account for biological biases of scRNA-seq data during pseudobulk simulation via scaling. For instance, normalization of read counts using housekeeping genes [50] or experimentally or computationally determined scaling factors that consider different mRNA content for individual cell types [44, 51, 52] were implemented to improve pseudobulk quality. These strategies constrain pseudobulk simulations to a limited set of cell types with available experimentally determined scaling factors. Dietrich *et al*. [44] have implemented such considerations in their above-mentioned bias-aware pseudobulk simulator SimBu.

Finally, after selecting cells given a ground-truth of cell-type proportions and an optional scaling step, the expression profiles of the cells are aggregated, i.e. read counts for each gene are summed across single cells (Fig. 2). In addition, the resulting total counts can be scaled up to counts per million or remain as raw counts representing the final pseudobulk sample.

While the approaches toward pseudobulk simulation from scRNA-seq data vary in detail (e.g. single cell sampling or data normalization), they fundamentally employ the aggregation of read counts of individual cells to obtain pseudobulk profiles (assuming a linear projection between single-cell and bulk space).

### Further considerations for pseudobulk quality

Using this pseudobulk simulation, several deconvolution tools have been trained or benchmarked [17, 20, 46–48]. However, looking at the above-mentioned (dis)similarities of pseudobulk and “real” bulk [47], one may argue that the performance of current simulators may not be perfect and pseudobulks display significant differences from real bulks that can affect their downstream applicability.

To understand these differences, it is important to consider the distinct steps of pseudobulk simulation. The scRNA-seq data input is usually already cell-type-annotated or even consists of multiple integrated datasets [44]. As mentioned above, from a technical perspective, scRNA-seq data are sparse [53, 54], which specific tools for single-cell data aim to eradicate via preprocessing focussing on denoising, imputing dropouts, clustering, cell-type annotation, or batch effect reduction [22–24, 34–37, 55]. To our knowledge, these tools are not yet routinely used prior to pseudobulk simulation. Other studies have included the addition of Gaussian noise to deal with inherent biases and zero inflation of scRNA-seq data or only use a subfraction of genes for simulation [46]. Overall, normalization of scRNA-seq data prior to pseudobulk simulation is an important aspect to consider. Typically, single-cell expression profiles are used as raw counts or normalized as counts-per-million or counts-per-10k reads (CP10K) prior to simulation. A recent study presented an alternative approach that accounts for the variation in the transcriptome sizes of different cell types using “Count based on Linearized Transcriptome Size” (CLTS) [56]. This method demonstrated enhanced performance in DE analysis across pseudobulks (81.9% of DE genes identified after CLTS normalization vs. 56.7% after CP10K normalization of scRNA-seq data) and in deconvolution (near perfect correlation between inferred and ground-truth proportions, i.e. *R* = 1.000).

Moreover, a linear combination of single-cells to simulate bulk data may seem imperfect, in particular when considering the inherent differences between bulk and scRNA-seq [48]. Even though pseudobulk simulation may reduce sparsity of single-cell data by aggregating single-cells across cell types with zero inflation for partially unique sets of genes, it generally propagates the nature of scRNA-seq data including dropouts or sparsity compared with bulk RNA-seq data. Also, deconvolution tools were found to perform better on pseudobulks than their corresponding bulk counterparts [38, 57]. This may be the result of pseudobulk-based training or the use of an scRNA-seq reference, or both. These factors introduce a bias toward the read count distribution of scRNA-seq data rather than that of “real” bulk data. Hu and Chikina [38] have reported a pseudobulk simulator that accounts for the same biological variance observed in bulk RNA-seq data by using scRNA-seq data derived from the same individual. They considered this approach to resemble more realistic bulk simulation settings. The resulting pseudobulk samples could only be less accurately deconvolved than pseudobulks generated by common simulations.

In conclusion, convolution as defined in the holistic transcriptome model is currently not performed in most cases, as it requires the usage of experimentally determined cell-type proportions to predict a bulk expression profile of the corresponding tissue. Rather, cell-type proportions are modeled, based on assumptions or randomly generated to simulate pseudobulks. Pseudobulk simulation mainly employs single-cell aggregation. We note that—to the best of our knowledge—there are no benchmarks that evaluated or compared pseudobulk simulators with respect to quality, e.g. correlation to actual matched bulk samples. Pseudobulk simulation strategies have been merely compared with regard to the single-cell sampling strategy, i.e. accounting for biological or technical variance, and their ability to push deconvolution tools to their limits [38]. Future efforts may be directed at implementing actual convolution, finding alternative simulation strategies and applying quality control focused on the comparison with matched bulk samples.

## Deconvolution of bulk RNA-seq data

**Figure 3:**
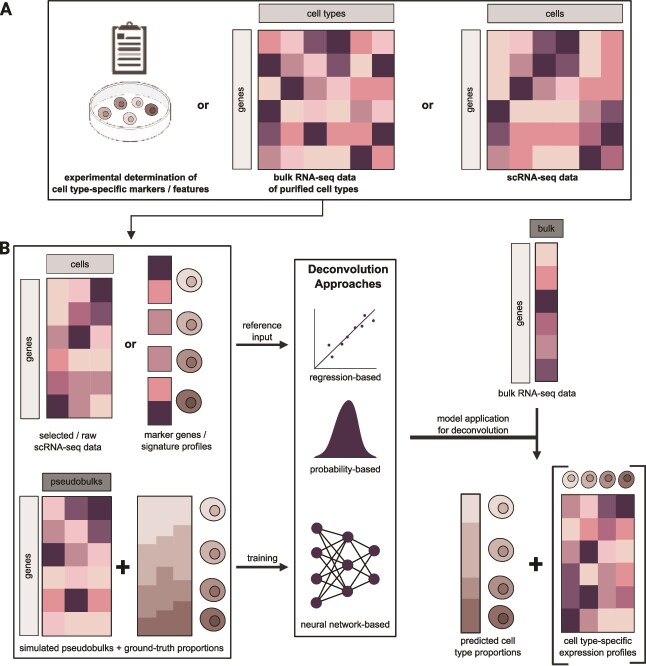
**Conceptual strategy employed for deconvolution of transcriptomics data.** (A) Variety of data inputs for existing deconvolution tools encompassing cell-type-specific marker genes, bulk RNA-seq data of purified cell types, or sc/snRNA-seq data. (B) Typical reference-based and probability/Bayesian-based deconvolution tools accept as input bulk RNA-seq data for deconvolution and markers or signatures of cell types or entire scRNA-seq datasets with cell types assumed to be present in the bulk data. Neural network-based deconvolution tools are trained with scRNA-seq-based simulated pseudobulks before being used for deconvolution. The final output encompasses cell-type proportions (cell-type deconvolution) and—generated in some tools—cell-type gene expression profiles (single-cell deconvolution). Schematic elements in (A) were created in BioRender. Wolfram-Schauerte, M. (2025) https://biorender.com/aa53ilm

The presented, simplified (de)convolution model envisions a bi-directional projection between bulk and single-cell data (Fig. 1). Most efforts have focused on the deconvolution of bulk RNA-seq data. Deconvolution to obtain cell-type proportions and gene expression signatures (Fig. 3) has been a major focus in the last decade aiming to take advantage of enhancing the molecular resolution of bulk RNA-seq data.

Notably, bulk transcriptomes can be derived from both RNA-seq or microarray expression data. The latter are not the predominant focus of this review. The reader may find more information to dive into the technical details and application of “microarray deconvolution tools,” which have been primarily regression-based, here [31, 58–61].

When classifying currently available deconvolution methods, one can generally distinguish deconvolution methods by three factors—the algorithmic principle, the type of reference used, or the biological question that can be addressed by deconvolution. Herein, we focus merely on the algorithmic basis to classify deconvolution tools, as it both illustrates the “evolution” of deconvolution tools over the last decade and their similar as well as unique working principles. It has to be noted that our review of deconvolution tools here does not cover all existing methods. For additional perspectives, readers may find other reviews helpful, which, e.g. focus on mathematical considerations, or emerging challenges, or tools that are not included here [21, 30, 62, 63].

The selection of deconvolution tools that we present here focus mainly on regression-based, probability/Bayesian-based, and neural network-based tools, which require reference information. Also, we highlight a subset of increasingly relevant reference-free and semi-supervised methods, which employ prior information about the number of cell types or selected marker genes only. These methods mostly make use of matrix decomposition, scoring, or Bayesian techniques. Importantly, we intend to provide an overview of these deconvolution methods and therefore do not focus on comparing or evaluating their performance, which is available in several other reviews or benchmarks [20, 30, 38, 48, 57, 64, 65]. An overview about the tools reviewed in this article is given in Supplementary Table 1, which details deconvolution strategy, algorithmic principle, input and output of the tools as well as use cases and citation statistics.

Overall, the fundamental principles of the reviewed deconvolution algorithms are conceptually similar. For one, these methods require a reference for cell-type expression profiles. These can be based on experimentally determined cell-type markers, bulk RNA-seq of isolated (e.g. by **fluorescence-activated cell sorting (FACS)**) cells, or scRNA-seq data (Fig. 3A). These references are utilized to train or inform a machine learning algorithm for deconvolution depending on the method (Fig. 3B). For instance, scRNA-seq data (subsampled or specifically processed) or signature profiles derived or provided from the reference are often used as input for regression- and probability-based deconvolution tools. Neural networks are usually trained for deconvolution with scRNA-seq-derived pseudobulks (simulated as described above). Then, deconvolution of bulk RNA-seq data is performed generating inferred cell-type proportions and potential additional cell-type-specific expression profiles as output (Fig. 3B).

### Regression-based and related deconvolution tools

Regression-based deconvolution infers cell-type proportions and expression profiles from bulk transcriptomics data by solving a multiple regression problem in an over-determined system (i.e. more marker genes than cell types) (Fig. 1, Equation 1).

One of the first tools that paved the way for **cell-type deconvolution** was **CIBERSORT** [66]. It was initially developed for microarray data, but later applied to RNA-seq data. CIBERSORT adaptively selects marker genes from an integrated signature matrix for cell types. Based on citations CIBERSORT is still the most popular deconvolution tool (Supplementary Table 1).

In addition to CIBERSORT, other marker/signature-based techniques include **EPIC** (Estimating the Proportion of Immune and Cancer cells) [52] or **xCell** [67] (Supplementary Table 1). Both these deconvolution tools are informed by gene expression data from isolated cell types, e.g. various immune cells. Similar to CIBERSORT, EPIC performs linear regression, while xCell applies gene set enrichment analysis (GSEA) [68] to obtain enrichment scores for each cell type in each sample to estimate cell-type abundance [67]. Similar to CIBERSORT, quanTIseq focuses specifically on deconvolution of immune cell types using constrained least squares regression [50]. By allowing the sum of predicted cell-type proportions to be lower than 1, it is one of the few tools that can explicitly predict the proportion of unknown cell types, which are not contained in the reference signature matrix.

With the rise of single-cell sequencing technologies, deconvolution tools were developed to make use of scRNA-seq and later also snRNA-seq data as prior information on cell-type gene expression profiles. These tools have recently been branded as “second-generation deconvolution tools” [57].

**Bseq-SC** (bulk sequence single-cell deconvolution) extracts high-variance and cell type-unique genes from scRNA-seq data to generate a signature matrix for deconvolution with CIBERSORT [69]. **CIBERSORTx**—the further development of CIBERSORT—pursues a similar strategy but instead uses a signature matrix based on scRNA-seq data or bulk RNA-seq data of purified cell types [70] (Fig. 3).

Using scRNA-seq data, several additional regression algorithms have been employed for bulk RNA-seq deconvolution such as dampened weighted least squares (**DWLS**), ordinary least squares (**OLS**), or nonnegative least squares (**NNLS**) (reviewed in [71]). **DWLS**-based deconvolution was shown to be quite robust and insensitive to different normalization techniques [72]. **FARDEEP** (Fast And Robust DEconvolution of Expression Profiles) was developed specifically for immune deconvolution of the TME with the objective to display increased robustness against outliers [73]. **MuSiC** (Multi-subject Single Cell deconvolution) integrates scRNA-seq data from multiple subjects to determine cross-subject features [74]. This information is then harnessed to weight genes based on high or low variance enabling to robustly deconvolve various bulk RNA-seq datasets. **MuSiC2** builds on MuSiC by additionally accounting for healthy and diseased samples identifying stable genes across different conditions [75].

Among others, strategies to increase the robustness of regression-based deconvolution include normalization for mRNA content per cell type, as implemented in **ABIS** [51], or linear regression modeling per gene across samples and cell types, as followed, e.g. by **RODEO** [76]. In addition, enhanced marker selection is employed to improve the input cell-type signatures. For instance, **AutoGeneS** performs multi-objective optimization to select marker genes from scRNA-seq data and excludes co-linear genes of several cell types that may interfere with deconvolution [77]. **scEM** selects marker genes and classifies cells using the consensus of four different clustering methods for scRNA-seq data [78].

Finally, not only scRNA-seq data, but also snRNA-seq data can be used to differentiate cell types, which is especially useful when analyzing solid tissues or cryo-preserved samples [63, 79, 80]. To the best of our knowledge, **Bisque** is the only deconvolution tool that has been explicitly tested with snRNA-seq data [79]. It assumes that the cell-type proportions in single-nuclei data resemble the ground-truth and transforms bulk data to accommodate the gene expression distribution in the pseudobulk prior to deconvolution.

In conclusion, characteristics that distinguish regression-based deconvolution tools are the algorithm used to solve the regression problem, the source and curation of the cell-type gene expression reference, and the possible use cases (Supplementary Table 1).

### Probabilistic and Bayesian models for deconvolution

Probabilistic models for deconvolution employ, for instance, Bayesian inference or maximum likelihood estimation (MLE). In the former case, a prior distribution of cell-type proportions and cell-type-specific gene expression profiles is generated on the basis of scRNA-seq data. Then, given the bulk RNA-seq data, the posterior distribution of cell-type proportions and expression profiles is inferred. While regression models usually require data linearity [81], probabilistic models can assume different data distributions such as log-normal, which was reported to be more accurate [82].

Some available probabilistic deconvolution tools output cell-type proportions only, as highlighted in Supplementary Table 1. **DeMix** [58] outputs the tumor proportion of a sample or **RNA-Sieve** [83] performs MLE to infer cell-type proportions. Some of these and additional methods can jointly output cell-type proportions and gene expression signatures. **ISOpure/ISOpureR** [84, 85] extract the tumor proportion of a bulk sample and its corresponding expression profile. **DeMixT** [86] can infer the proportions of immune, stromal, and tumor cells and their expression profiles. Tools such as **BEDwARS** [87], **BayesPrism** [88], or **BLADE** [89] are more widely applicable, as they can infer proportions and gene expression profiles of many cell types in parallel using Bayesian inference (Supplementary Table 1).

Recently, a semi-supervised deconvolution tool termed **GLDADec** (guided Latent Dirichlet Allocation Deconvolution) has been developed [90]. It makes use of the natural language processing algorithm Latent Dirichlet Allocation (LDA), which was adapted to estimate cell-type proportions in bulk transcriptomics data similar to topics in text documents. To constrain the estimation of cell-type proportions, they integrated prior information of marker gene names that are associated with distinct cell types, which are regarded as topics (Supplementary Table 1).

### Neural network-based deconvolution

Deep learning via artificial neural networks (ANNs) also present a suitable model architecture for deconvolution, as ANNs may learn interconnections of genes and cell types better than “classical” regression techniques [45, 46, 91]. In order to train an ANN, larger amounts of bulk RNA-seq data with known ground-truth of cell-type proportions are required compared with, for instance, most regression-based methods. As such datasets are generally rare, researchers implemented pseudobulk simulations using scRNA-seq data for training. Thereby, the ground-truth of cell-type proportions is well defined for each simulated pseudobulk that can be used for supervised learning.

To the best of our knowledge, three ANN-based cell-type deconvolution tools have been developed to this day (see also Supplementary Table 1). For one, **Scaden** (single-cell assisted deconvolutional deep neural network) uses the 10 000 most variable genes for training and estimation of cell-type abundance [45]. It outputs the average prediction of three different multilayer perceptrons. **TAPE** (tissue-adaptive deconvolution) is based on an autoencoder architecture learning cell-type proportions in latent space through reconstruction of input samples [46]. In addition to a cell-type proportion output, TAPE also generates cell-type expression signatures. **NNICE** (Neural Network Immune Contexture Estimator) passes quantiles of bulk RNA-seq input through independent neural networks whose output is then integrated in a cell-type proportion estimate [91].

### Reference-free and semi-supervised deconvolution methods

Compared with most of the previously mentioned “second-generation” deconvolution methods that make use of (scRNA-seq) reference information, reference-free and semi-supervised methods usually do not require such data. Methodologically, these methods are based on regression, probabilistic models, or matrix factorization and decomposition approaches, as detailed in Supplementary Table 1. For instance, **DECODER** (*de novo* compartment deconvolution and weight estimation of tumor samples) infers major cell types (e.g. immune cells or tumor cells) from bulk expression data without prior information of marker genes [92]. Using nonnegative matrix factorization, it infers the cell-type proportions and their marker genes *de novo*. Other methods relying on matrix decomposition or regression include BisqueMarker [79], Deblender [93], Deconf [94], csSam [32], or TOAST [95]. Probabilistic models apart from GLDADec, DeMix, and DeMixT (described above) include CDseq [96], DebCam [97], or LinSeed [98], all of which do not require external reference information (Supplementary Table 1). Since these methods do not rely on single-cell expression information, they can be also applied in cases where high-quality scRNA-seq data for the tissue of interest are not available.

In conclusion, various machine learning tools for deconvolution have been developed based on different algorithmic principles. They differ not only in terms of input information for deconvolution, but also in their capability to adapt to various numbers of cell types in a given tissue. Their popularity and application is partially also reflected in their citations with CIBERSORT being the most popular tool (Supplementary Table 1). Moreover, our screening revealed that each deconvolution tool has been developed and tested using distinct sets of bulk and/or single-cell transcriptomics data (Supplementary Table 2). Based on the original publications, only few datasets have actually been used in the development, testing, or application of several deconvolution tools, which limits the comparability of these tools.

### Single-cell reference choice and annotation

“Second-generation” deconvolution methods have been shown to perform deconvolution of different ranges of cell types (Supplementary Table 1). This is influenced by the cell types of interest in the given bulk tissue as well as the single-cell reference. Thus, the annotation of the single-cell reference is a key factor for deconvolution, too. On the one hand, the confidence of the cell-type annotation can be a crucial factor; on the other hand, the granularity chosen for cell-type annotation is critical [99]. The finer the grain of annotation, the more low abundant cell types or subtypes in the bulk may be predicted that can challenge deconvolution tools due to lack of marker genes or simply the number of cell types to deconvolve [99]. Also, cell-type annotations can differ between snRNA-seq and scRNA-seq [100], which is important to consider for the choice of reference type.

The single-cell reference can be derived from a single dataset or from multiple datasets integrated across different individuals, batches, or studies. This often includes single-cell atlases that offer insights into the transcriptomic profiles of up to millions of cells [101]. However, scRNA-seq data integration, e.g. in form of atlases, can come with several limitations when using “second-generation” deconvolution tools with single-cell references. Using replicates from different sources, including different donors, library preparation, and sequencing platforms or even (disease) conditions, unexpected variations may occur during deconvolution of bulk transcriptomes. As detailed by Maden *et al*. [63], cell scales are usually lost in atlases, as the single-cell normalization is applied relative to total mRNA per cell. Also, some cell types may be poorly distinguished across datasets. SCCAF-D takes this limitation into account by identifying the most representative cells per cell type across datasets for single-cell reference creation that is then used for downstream deconvolution [102].

### Application of deconvolution techniques

A major fraction of current deconvolution tools has either been designed for or assessed in the context of cancer, for instance, to deconvolve the TME, metastatic cancers, or to dissect bulk data into cancer and non-cancer cells [103–105] (see also Supplementary Tables 1 and 2). Kang *et al*. [106] applied CIBERSORTx using matched single-cell and bulk RNA-seq data of gastric tumors to study the TME, thereby discovering patient subgroups that show TME-dependent treatment response. CIBERSORT(x)-based deconvolution was also employed to specifically analyze the immune cell fractions in various cancer samples including melanoma and ovarian cancer datasets [47, 107].

CIBERSORT has also been applied in other contexts. Deconvolution with CIBERSORTx helped to identify changes in the immune cell-type abundance and the overall immunesuppression in sepsis patients [108] and to characterize cell-type changes in the aging mouse brain [109], the human brain [110], and placenta [111]. In metabolic-associated fatty liver disease, researchers applied MuSiC to record changes in cell-type proportions during disease progression, which indicated disease-associated gene expression signatures [112]. Kim *et al*. [113] deconvolved bulk RNA-seq data of alcohol-associated hepatitis to discover significant changes in cell-type abundance, which align with the severity of the disease. In Alzheimer’s disease research, deconvolution of bulk transcriptomes using snRNA-seq-based reference profiles was applied to determine cell-type proportions in various brain regions [114]. These proportions explain changes in gene expression in Alzheimer’s disease that originate from altered cell-type proportions and concordantly changed marker gene contribution. Also, higher basal monocyte activation in patient blood—as identified by deconvolution—was found to reduce susceptibility to influenza A virus infection [93].

These examples demonstrate the need and value of robust deconvolution methods in various areas of basic and biomedical research. It has to be noted that our choice of the above-mentioned examples may not be representative of the diverse deconvolution applications in “real-world” research examples.

## Evaluation and benchmarks

For the evaluation of deconvolution tools several main strategies are available, which include the usage of the following dataset types:

1. pseudobulks simulated on the basis scRNA-seq data (also referred to as *in silico* framework);
2. mixtures of bulk RNA-seq data of purified cell types or cell lines (also referred to as *in vitro* framework);
3. datasets with scRNA-seq and bulk RNA-seq samples from the same tissue and ideally same individual;
4. datasets of bulk RNA-seq data and matched cell-type abundance measurements (e.g. flow cytometry or IHC) from the same individual (also referred to as *in vivo* framework);
5. “matched” datasets with bulk RNA-seq, scRNA-seq, and cell-type abundance measurements from the same tissues and individuals—the ideal datasets.

Each of these dataset types are important for the development and evaluation of deconvolution methods. However, as deconvolution is also frequently applied to stand-alone bulk RNA-seq data from large cohorts, where orthogonal measurements of scRNA-seq and cell-type abundance are usually lacking, dataset types including bulk RNA-seq data are particularly important for deconvolution evaluation. Specifically, matched datasets including bulk RNA-seq, scRNA-seq, and abundance measurements stand out as ideal datasets. Such datasets enable extensive assessment of deconvolution tools based on bulk and pseudobulk samples with a joint scRNA-seq reference. Notably, they are similarly important for the evaluation of convolution tools, as cell-type proportions are key to simulate pseudobulk samples from scRNA-seq data matched to the target bulk RNA-seq samples.

### Datasets for (de)convolution

**Figure 4:**
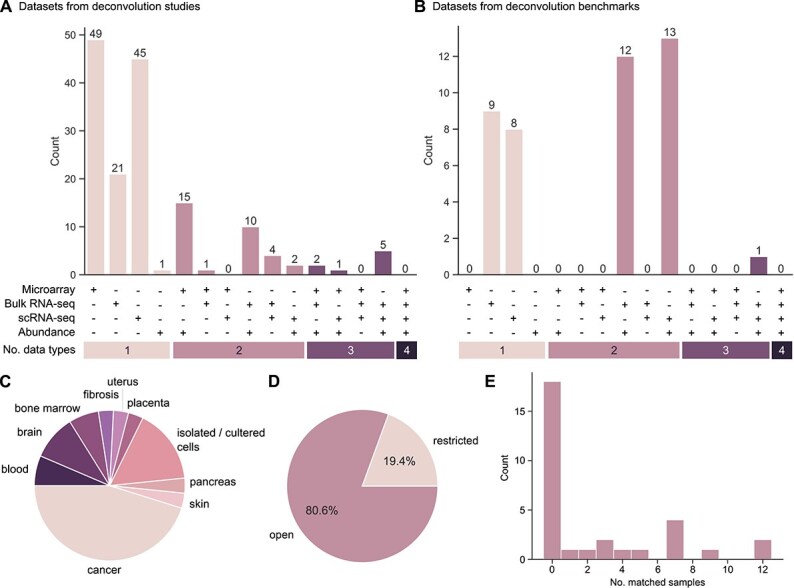
**Datasets used and available for deconvolution evaluation.** (A, B) Barplots representing the number of unique datasets including one, two, three, or four data entities (microarray, bulk RNA-seq, scRNA-seq, abundance) used in deconvolution methods papers (A) and deconvolution benchmarks (B). Original data presented in Supplementary Tables 2 and 4, respectively. (C–E) Results of an independent search for datasets suitable for convolution and deconvolution; original data presented in Supplementary Table 5. Pie charts indicate fractions of datasets with specific tissue-association (C) or restricted data access (D). The barplot represents distribution of matched samples, where scRNA-seq and bulk RNA-seq data are generated from the same biospecimen, across identified datasets (E).

To obtain an overview of the availability and usage of above mentioned dataset types in the development and testing of deconvolution tools, we gathered information about datasets used in the reviewed deconvolution methods papers summarized in Supplementary Table 2. Our analysis clearly showed that the majority of datasets includes only one entity (e.g. microarray, bulk RNA-seq, or scRNA-seq) (Fig. 4A). This indicates the frequent use of stand-alone bulk RNA-seq or microarray data for gaining novel insights, when developing deconvolution methods, or scRNA-seq data serving as a reference or for pseudobulk simulation for deconvolution. Microarray and bulk RNA-seq with matched abundance measurements are employed as dataset types composed of two entities, but show reduced availability or usage (see also Supplementary Table 2). Ideal “matched” datasets are rarely used or available with only five datasets matching the criteria of bulk RNA-seq, scRNA-seq and abundance measurements (Fig. 4A). We extended our search to deconvolution benchmarks addressing mainly “second-generation” deconvolution tools that are not primarily built or tested on microarray data (Supplementary Table 3). It stands out that the analyzed benchmark studies primarily used only one-entity and two-entity datasets (Supplementary Table 4, Fig. 4B). As expected, datasets composed of either scRNA-seq and bulk RNA-seq data alone or additionally with corresponding abundance measurements were predominantly used in these benchmarks (Supplementary Table 4). However, no dataset including matched bulk and scRNA-seq was found to be used in a deconvolution benchmark, while only one ideal matched dataset was utilized (Fig. 4B). Overall, this underscores the underrepresented usage of matched datasets in deconvolution methods papers and benchmarks, which we think are crucial for their evaluation and comparison. Moreover, it highlights again that benchmarking and evaluation of deconvolution methods is mostly based on the *in silico* framework. However, while the abundance of high-quality benchmarking datasets is limited, the benchmarks used consistent data input, e.g. via best-practices regarding normalization and formatting, to allow for comparability of the deconvolution tools.

Due to the observed underrepresentation of matched datasets, we searched the Gene Expression Omnibus (GEO) for human tissue datasets containing bulk RNA-seq and scRNA-seq samples of the same biospecimen (see Methods). Specifically, we applied the following GEO search term logic: (“single-cell transcriptomics” OR “scRNA-seq” OR “single-cell RNA sequencing”) AND (“bulk RNA sequencing” OR “RNA-seq”) AND (“matched” OR “paired” OR “same donor”) AND “Homo sapiens.” A total of 31 of the 176 hits of our search qualified as datasets composed of both bulk RNA-seq and scRNA-seq data from human tissue (Supplementary Table 5). The majority of those were derived from cancer samples followed by isolated or cultured cells and samples from brain and blood (Fig. 4C). For 19.4% of the datasets, access restrictions applied due to privacy concerns, which one might thus regard as unsuitable for evaluation or benchmarking of deconvolution methods (Fig. 4D). Finally, we aimed at quantifying whether and how many of the bulk RNA-seq and scRNA-seq samples are matched (derived same tissue and same individual). This analysis is *per se* challenging, as the annotation of the “matching” of scRNA-seq and bulk RNA-seq samples is in many cases lacking in both GEO description and the original publication (Fig. 4E). Only in fewer cases (10 datasets), datasets with at least three matched samples matching our criteria were identified with confidence (Fig. 4E). Notably, we could not obtain information whether these datasets contain additional cell-type abundance measurements. Overall, this underlines the lack of suitable matched datasets for evaluating and benchmarking (de)convolution and pseudobulk simulation tools.

### Benchmark-based challenges for deconvolution

Apart from the availability of suitable datasets, there are several other important considerations for deconvolution performance. In this regard, readers may also find important benchmark articles [20, 30, 38, 48, 57, 64, 65] or reviews on current challenges in deconvolution [63] useful. Here, we will highlight a few examples of important features of and demands on deconvolution tools that these benchmark studies have addressed previously. Notably, the benchmarks investigated different sets of deconvolution tools alongside different biological contexts (e.g. tissues and/or diseases) (Supplementary Table 3).

For one, dealing with low abundant cell types—those are often the most interesting, as they can change upon disease progression—is a critical feature, which varies depending on the deconvolution tool, cell type and tissue studied [20, 38, 48, 57, 64, 65]. Moreover, missing a cell type in the reference is detrimental for deconvolution, as it distorts the estimated cell-type proportions [20, 57]. The recently developed deconvolution tool SECRET can adapt to missing cell types in the reference [105]. In general, estimating unknown content in deconvolution is a problem barely addressed. Few tools, such as quanTIseq [50], provide estimates for unknown cell types/content.

Deconvolution methods require differently normalized input data, e.g. linear, logarithmic, variance stabilizing, or square-root transformed, as illustrated in Supplementary Table 1. The impact of normalization on deconvolution has been benchmarked, showing that linear transformations generally perform the best [20] or corrections of varying library sizes are essential for deconvolution performance [63, 64]. This may also explain the improved deconvolution using CLTS normalization—also a linear transformation—of single-cell data [56]. In general, normalization approaches aim to maintain or transform single-cell and bulk data to comparable scales given their technical differences [63].

Data normalization is also related to the handling of batch effects between single-cell and bulk transcriptomes and represents an important aspect of deconvolution methods [63]. Consequently, various strategies have been established to reduce the effects of biological or technical variance. Among others, MuSiC2 removes genes that exhibit effects specific to distinct conditions, allowing for deconvolution under various (clinical) conditions [75]. Bisque learns bulk data transformations to reduce variation of bulk and single-cell data [79]; SCDC uses the ensemble of deconvolution results from various single-cell references of different origins or conditions to handle batch effects [115]. Further, with the advent of single-cell transcriptomic foundation models, new approaches are available to integrate single-cell data from diverse batches that could help advance batch effect handling in future efforts [116, 117].

Moreover, the applicability of deconvolution tools has been a major focus. Some tools infer actual cell-type proportions, while others generate arbitrary scores for cell types or “compartments.” Importantly, the usage of such scores is limited to comparisons of cell-type proportions with cell types within a sample or within a cell type, but restricts generalized comparisons across samples. In addition, some deconvolution tools are designed for specific tissues or cell types, e.g. blood/immune cells [48]. Hence, deconvolution tool applicability is not only depending on the algorithm, but also the specific biological question or tissue of interest. This complicates appropriate choices by the users.

Also, to perform user-friendly custom evaluations and apply a variety of deconvolution tools, several benchmarks created pipelines to provide access to multiple deconvolution methods [48, 57, 65]. Such studies identified MuSiC, DWLS, CIBERSORT, Scaden, and BayesPrism among the top-performing tools for specific tissues or use cases.

Finally, an important consideration for evaluation and benchmarking is the interplay of pseudobulk simulation—as a form of convolution—and deconvolution methods via the *in silico* framework. In the CATD (critical assessment of transcriptomic deconvolution) benchmark, it was observed that cell-type deconvolution is very sensitive to the simulation method creating the pseudobulk [65]. Also, the ANN-based deconvolution tool Scaden was found to be particularly accurate when using PBMC pseudobulk data as input [57]. Interestingly, the “real” bulk with the same cell-type proportions (*in vivo* framework) was less accurately deconvolved by Scaden. Considering that the ANN in Scaden is trained with pseudobulks derived from the scRNA-seq reference prior to deconvolution [45], enhanced performance on pseudobulk data does not come as a surprise, but highlights the inherent limitations of the abundant *in silico* framework.

On the other hand, to the best of our knowledge, convolution or pseudobulk simulation benchmarks, respectively, are lacking in the field. Convolution as defined in the holistic transcriptome model is rarely applied and the correlation of predicted bulks to the corresponding bulks is usually not assessed. Pseudobulk simulations have also not been benchmarked and often their use and performance are justified based on successful deconvolution of “real” bulk samples after pseudobulk-based training.

Overall, it is striking that the number of available deconvolution tools (Supplementary Table 1) exceeds the number of available or employed benchmarking datasets (Supplementary Tables 2 and 4, Fig. 4). Most of the above-mentioned benchmarks merely employed the *in silico* framework. Considering the performance of pseudobulk simulators and the enhanced deconvolution performance on pseudobulks [57], a paradigm shift in simulation approaches, e.g. toward actual convolution, and benchmarking frameworks may offer promising directions for more comprehensive benchmarks. This may not only mean generation of more data for the *in vitro* and *in vivo* frameworks, but also enhancing the quality of the *in silico* framework, e.g. pseudobulk simulators or creation of convolution methods.

## A holistic transcriptome model to accomodate convolution and deconvolution

Our review has highlighted that while convolution or currently employed pseudobulk simulation plays a critical role in training and assessment of deconvolution methods, both directions were so far mainly considered separately. The apparent focus in the community lies on deconvolution—due to the vast availability of bulk RNA-seq data and the opportunity to gain significant biomedical insights through their deconvolution. We envision a “holistic transcriptome model” that takes the interconnection of convolution and deconvolution into account—basically, a circular model considering their mutual interplay. Conceptually, this model extends beyond deconvolution by stressing the additional importance of convolution, where advances in one direction can directly benefit the improvement of the other. Moreover, we stress the importance of actual convolution approaches compared with currently employed pseudobulk simulations. This model must not necessarily adhere to the simplified idealistic mathematical model we introduced. It could implement the general concept and integrate the learnings from both convolution and deconvolution tools [21, 62, 63]. We note that, considering previous benchmarks, limitations such as handling missing cell types or estimating unknown content might be valuable to consider in order to improve deconvolution tools. Overall, we envision a holistic transcriptome model to perform convolution and enhanced deconvolution for instance by learning projections between bulk and single-cell data. Several recent developments in this area indicate promising leaps toward such a model.

For instance, the recently published tool **scSemiProfiler** considers this aspect by learning transformation between single-cell and bulk RNA-seq data with a variational autoencoder [118]. This tool currently works with matched datasets where a patient cohort is screened by bulk RNA-seq and representatives of cohort subgroups are then selected to perform scRNA-seq. Furthermore, **ReDeconv** approaches the improvement of pseudobulk generation by normalizing scRNA-seq data based on average transcriptome sizes of cell types (CLTS normalization) [56]. Using CLTS-normalized scRNA-seq data for pseudobulk simulation, they showed improved performance of their probabilistic deconvolution model. From our point of view, these great developments indicate a promising direction, where a holistic transcriptome model may go to in the future.

To advance the current (de)convolution toolkit and methodology toward a truly holistic transcriptome model, we highlight two key areas for improvement: data frameworks and the interplay between convolution and deconvolution.

### Advances of data frameworks

For one, data frameworks represent a key area for improvement of (de)convolution. Many datasets lack at least one of the three modalities included in the holistic transcriptome model—often cell-type proportion measurements are lacking. The ideal matched dataset includes bulk RNA-seq, scRNA-seq, and cell-type abundance data for an optimal evaluation setting and is generally rare. More high-quality matched datasets of various tissues, conditions, and/or individuals are thus required to develop and evaluate a holistic transcriptome model. One may also consider running single-cell multi-omics analysis of the same samples to increase the availability of orthogonal measurements for the benchmarking of tools for (de)convolution, pseudobulk simulators, and in other domains. For instance, modalities such as spatial transcriptomics with single-cell resolution can be used to determine the ground-truth of cell-type proportions for bulk RNA-seq deconvolution datasets. However, matched datasets are cost- and time-intensive to produce. Consequently, also more datasets with two modalities or data of the *in vitro* framework can be of great value. Notably, when performing large cohort studies, it might be challenging to select suitable candidates for multi-omics screening, while all candidates are be routinely assessed by bulk RNA-seq. It is key to select most representative candidates for single-cell analysis, for instance, to properly cover the variance across individuals and conditions with the additional single-cell data. Recently developed tools such as scSemiProfiler will be able to assist the process of generating matched datasets suitable for large cohort analysis [118].

Beyond increasing data availability, establishing consensus, standardized benchmark datasets is critical. Dedicated repositories such as DeconvData [57] could provide a central resource, hosting datasets across all three frameworks (*in silico*, *in vitro*, *in vivo*) to enhance comparability across studies. Moreover, the curation of the single-cell reference information used for deconvolution is a critical aspect [38]. Uniform and standardized analysis and processing of sc/snRNA-seq data such as employed in Sfaira [119], CellxGene [120], or the Human Cell Atlas [101] could be of great benefit to increase data quality, access, and inter-study comparability.

So far, deconvolution focussed primarily on human and—to a lesser extent—on mouse transcriptomic data. Given the abundance of single-cell transcriptome data for these organisms, deconvolution tool application was focused on these organisms, while application to other eukaryotic species is limited. Altogether, we envision that advancements in data generation, accessibility, and unified processing will impact all areas of the holistic transcriptome model: actual convolution, deconvolution, and their evaluation and benchmarking. Further, these data-driven considerations for a holistic model may also shape future experimental designs.

### Interplay of convolution and deconvolution

Second, it will be key to more profoundly consider both directions of the holistic transcriptome model, which represents the key advantage of the comprehensive perspective we suggest with this model. It needs to be emphasized that convolution and deconvolution are interdependent operations when it comes to their assessment and partially also training. Deconvolution tools are trained and evaluated with pseudobulks simulated with a convolution-like approach, while pseudobulk simulation is often evaluated on how well the resulting data can be deconvolved.

Since deconvolution tools are often trained and validated on pseudobulks generated through a convolution-like process, their evaluation inherently depends on the quality of the pseudobulk simulation model. Thus, developing actual convolution techniques further improving pseudobulk simulators—such as optimizing normalization strategies [56] or leveraging gene subsets to enhance bulk-pseudobulk correlation—could directly improve deconvolution accuracy. Additionally, reconstructing bulk transcriptomes from single-cell references and cell-type proportions inferred by deconvolution could serve as an internal validation step, closing the loop between convolution and deconvolution.

Given the number and diversity of deconvolution tools (Supplementary Table 1), it may be challenging to keep track of their availability, strengths, and bottlenecks. Continuous benchmarking efforts may also contribute to more clearly define applicabilities and limitations of existing deconvolution tools [20, 30, 38, 48, 57, 64, 65]. This will add to a comprehensive user’s guide to deconvolution and possibly even actual convolution tools in the future. Unified benchmarking datasets of *in silico*, *in vitro*, and *in vivo* framework—especially including matched datasets—may present as one important future resource in the field (also discussed in [63]).

Advancing data generation, accessibility, and unified processing will strengthen all aspects of the holistic transcriptome model—improving both convolution, also pseudobulk simulation, and deconvolution while refining evaluation and benchmarking strategies. These efforts will not only enhance methodological robustness, but also expand the applicability of transcriptomic analysis in both basic and translational research.

## Conclusion

In this review, we discussed convolution and deconvolution, their interconnectivity, and focused in particular on related pseudobulk simulation and deconvolution tools. We highlighted the principles of pseudobulk simulation—and its differences to actual convolution—and extensively reviewed the variety of deconvolution tools focussing on their strategy for deconvolution, applicability, and evaluation. Our review and analysis identified that available datasets stand out as a key bottleneck alongside quality assessment for pseudobulk simulators as well as the lack of actual convolution approaches. Moreover, we suggested a framework to improve the diverse efforts of past, present, and future to design convolution-like pseudobulk simulators and deconvolution methods toward a holistic transcriptome model, which distinguishes itself from existing efforts with a novel perspective considering the mutual interplay of both convolution and deconvolution.

Not only may more datasets be required, but also novel data entities might be integrated. Spatial transcriptomics with single-cell resolution, for instance, is directly informing about spatial context and gene expression therein and thereby provides information on cell-type abundance, too. There is already an abundance of spatial deconvolution methods such as SPOTlight [121], Tangram [122], or Cell2location [123], some of which are bundled in spacedeconv [124]. In spatial deconvolution, spots are usually treated as miniature pseudobulks that can be deconvolved with single-cell data. These pseudobulks might differ from single-cell-based pseudobulks discussed here—also due to technical differences between measuring transcriptomes of spots and single cells. Vice versa, insights from bulk transcriptome deconvolution—as outlined here—may also stimulate the further development of deconvolution methods in the spatial transcriptomics field. Also, other omics entities may play roles to enhance (de)convolution. For instance, deconvolution also finds application in the field of DNA methylation [125]. It would be exciting to combine deconvolution across multiple omics layers, such as the methlyome and transcriptome, and compare their performance. Advances in these areas will have a huge impact on efforts to build a holistic transcriptome model, wherein deconvolution tools can robustly analyze datasets across tissues, diseases, and patients, and actual convolution tools or simulators can reliably generate authentic pseudobulk transcriptomes.

Recent developments in machine learning may also boost the development of a holistic transcriptome model. Natural language processing and transformer models have become popular due to their outstanding performance [116, 117]. Now, scientists make use of them in the field of transcriptomics, outperforming state-of-the-art tools in various aspects including scGPT, scFoundation (both trained on scRNA-seq data), or BulkRNABert (trained on bulk RNA-seq data) [126–128]. Incorporating such recent developments in the field of single-cell omics into the holistic transcriptome model has the potential to boost (de)convolution applications with important implications for the design of future studies that involve multi-omics technologies for molecular disease understanding.

## Methods

Literature was searched via Google Scholar, Web of Science, and PubMed. Citation statistics were retrieved from PubMed at the given date indicated in Supplementary Table 1. Datasets used in the deconvolution tool manuscripts and benchmark studies were identified from the paper and details (accessions, content, etc.) were retrieved from the original publication and dataset repository (GEO, ENA, SRA, and others). Research results are available in Supplementary Tables 1–4.

Search for matched datasets (bulk and scRNA-seq) was performed with GEO Dataset Search on 6 February 2025 using the following terms: (”single-cell transcriptomics” OR ”scRNA-seq” OR ”single-cell RNA sequencing”) AND (”bulk RNA sequencing” OR ”RNA-seq”) AND (”matched” OR ”paired” OR ”same donor”) AND ”Homo sapiens.” Datasets without bulk or single-cell RNA-seq data were removed as well as those including organisms other than human. Final results are available in Supplementary Table 5.

**Key Points**

- We overview deconvolution and convolution methods linking bulk and single-cell transcriptomics within a holistic transcriptome model.
- We highlight the functional principles of current pseudobulk simulators and their deviation from true biological convolution.
- We analyze the diversity and functionality of existing deconvolution tools, with a comprehensive summary of tools and datasets.
- The lack of suitable datasets remains a major bottleneck for (de)convolution research.
- We synthesize concepts to drive advances within the holistic transcriptome framework.

## Supplementary Material

Supplementary_Tables_bbaf388

## Data Availability

All data associated with this manuscript is provided with the manuscript itself and the Supplementary Tables. Links or GEO identifiers for mentioned datasets are provided with the Supplementary Tables.
